# Physical Hydrogels of Oxidized Polysaccharides and Poly(Vinyl Alcohol) for Wound Dressing Applications

**DOI:** 10.3390/ma12091569

**Published:** 2019-05-13

**Authors:** Raluca Ioana Baron, Madalina Elena Culica, Gabriela Biliuta, Maria Bercea, Simona Gherman, Daniela Zavastin, Lacramioara Ochiuz, Mihaela Avadanei, Sergiu Coseri

**Affiliations:** 1 “Petru Poni” Institute of Macromolecular Chemistry of Romanian Academy, 41 A, Grigore Ghica Voda Alley, 700487 Iasi, Romania; culica.madalina@icmpp.ro (M.E.C.); biliuta.gabriela@icmpp.ro (G.B.); bercea@icmpp.ro (M.B.); mavadanei@icmpp.ro (M.A.); 2 Faculty of Pharmacy, “Grigore T. Popa” University of Medicine and Pharmacy Iasi, 16th University Str., 700115 Iasi, Romania; simonagherman08@yahoo.com (S.G.); daniela.zavastin@umfiasi.ro (D.Z.); ochiuzd@yahoo.com (L.O.)

**Keywords:** oxidized cellulose, oxidized pullulan, poly(vinyl alcohol), hydrogel, self-healing, cytotoxicity

## Abstract

Two natural polymers, i.e., cellulose and water soluble pullulan, have been selectively oxidized employing the TEMPO-mediated protocol, to allow the introduction of C_6_-OOH groups. Thereafter, the composite hydrogels of poly(vinyl alcohol) (PVA) and different content of the oxidized polysaccharides were prepared by the freezing/thawing method. The Fourier transform infrared spectroscopy (FTIR) has been used to discuss the degree of interaction between the hydrogels constituents into the physical network. The homogeneity of the prepared hydrogels as revealed by the SEM show an excellent distribution of the oxidized polysaccharides inside the PVA matrix. The samples exhibit self-healing features, since they quickly recover the initial structure after being subjected to a large deformation. The cell viability was performed for the selected hydrogels, all of them showing promising results. The samples are able to load L-arginine both by physical phenomena, such as diffusion, and also by chemical phenomena, when imine-type bonds are likely to be formed. The synergism between the two constituents, PVA and oxidized polysaccharides, into the physical network, propose these hydrogels for many other biomedical applications.

## 1. Introduction

Hydrogels are remarkable polymeric networks classified as: “two- or multicomponent systems consisting of a three-dimensional network of polymer chains and water that fills the space between macromolecules” [1]. Due to their intrinsic properties, and their sensitive behavior to the fluctuations of the environment stimuli, hydrogels are extensively used as biomaterials exhibiting excellent biocompatibility. They can be used as scaffolds, providing structural integrity to tissues, controlled release of drugs and proteins to tissues. Moreover, they could serve as adhesives between tissue and material surfaces. Therefore, hydrogels’ properties are crucial for tissue engineering as well as other applications in the biomedical field. Properties such as swelling behavior, mechanical strengths and biocompatibility are drastically dictated by the nature of the constituents. Synthetic hydrogels include poly(vinyl alcohol) (PVA), poly(ethylene glycol), poly(vinyl pyrrolidone), or poly(acrylic acid). They have the advantages of easiness of processing and a wide versatility of mechanical and chemical properties. Natural hydrogels show indisputably preferences when biodegradability, biocompatibility and superior cell adhesion properties are required. Proteins and polysaccharides are the two most important classes of natural polymers used in hydrogels fabrication. In the last years, a combination between synthetic and natural polymers has been increasingly used for the hydrogel preparation in order to combine the advantages of the two original polymer classes. Polysaccharides especially have gained a lot of consideration due to their abundance and renewability, being used in their native form or as a specific derivative. For example, the oxidation of polysaccharides represent one of the most suitable approaches to introduce new functionalities, i.e., aldehyde, ketone or carboxylic [2,3,4,5], that are able to serve for further derivatisation or as anchoring sites of different molecules, broadening the applications area of these products [6,7,8,9]. In our very recent studies, we have reported the preparation and characterization of oxidized cellulose (or oxidized pullulan)—PVA hydrogels with different content of oxidized polysaccharides [10,11]. Aiming to further explore the usefulness of these versatile materials, in this paper we present new findings on the synthesis, characterization and application of these hydrogels, for wound dressing applications. The main advantages when using the oxidized cellulose or pullulan for the hydrogels preparation, are: i) Water is used as a sole solvent for both components: Oxidized polysaccharide and PVA, avoiding the use of complicated solvent mixtures required by the unoxidized cellulose, and ii) due to the extremely high number of COOH groups incorporated after oxidation, the oxidized polysaccharide component takes over the role of crosslinking agent, not requiring the presence of an additional, often toxic reagent. To test the efficiency of the synthesized hydrogel for drug loading and release, we propose the use of L-arginine, because it is an amino acid involved in the synthesis of collagen, re-epithelialization and tissue reorganization [12,13,14]. 

## 2. Materials and Methods

### 2.1. Materials

Poly(vinyl alcohol), molecular weight M_w_ of 8.9 × 10^4^–9.8 × 10^4^ g/mol, 99% hydrolized, Avicel^®^ PH 101 purified microcrystalline cellulose, were purchased from Sigma-Aldrich (Vienna, Austria). Pullulan, M_w_ = 1.5 × 10^5^ g/mol (TCI Europe, Bruxelles, Belgium) was dried overnight under vacuum at 100 °C prior to use.

2,2,6,6-Tetramethyl-1-piperidinyloxy (TEMPO), sodium bromide, 9% (wt.) sodium hypochlorite and other chemicals and solvents were of pure grade (Sigma Aldrich), and used for pullulan oxidation without further purification.

Cytotoxicity tests were done using the following materials and reagents: Dulbecco’s Modified Eagle Medium (DMEM), which comprise of: 4500 mg/mL glucose, 110 mg/L sodium pyruvate and 0.584 mg/L L-glutamine); Bovine Fetal Serum (BFS), heat inactivated, non-USA origin, sterile-filtered; Penicillin/Streptomycin/Neomycin (P/S/N) solution, (5000 units penicillin, 5 mg streptomycin and 10 mg neomycin/mL), sterile-filtered; Phosphate Buffered Saline (PBS) solution, sterilised, suitable for cell culture); 3-(4,5-Dimethyl-2-thiazolyl)-2,5-diphenyl-2H-tetrazolium bromide, (MTT).

N^ω^-nitro-L-arginine methyl ester hydrochloride (NO_2_-Arg-OMe, ≥98%, M_W_ = 269.69). All other chemicals and reagents were from the analytical grade. The in vitro drug release studies were realized on a spectrophotometer Specord 250, Analytic Jena (Jena, Germany). All materials and reagents were purchased from Sigma-Aldrich unless otherwise mentioned.

### 2.2. Preparation of Oxidized Polysaccharides

Cellulose (Avicel^®^ PH 101) and pullulan were used to prepare 6-carboxy corresponding counter partners. The protocol involves the presence of TEMPO (0.5 mmol/g polysaccharide), NaBr (8 mmol/g polysaccharide) and NaClO (8 mmol/g polysaccharide), at room temperature and pH = 10. The detailed information could be found in a previous work [8].

### 2.3. Preparation of the Composite Hydrogels

Oxidized polysaccharides, i.e., cellulose (OxC) or pullulan (OxP) solutions of 5% were prepared by dissolving the dry samples in Millipore water under vigorous magnetic stirring, 2 h at room temperature. A solution of 5% PVA was prepared dissolving PVA at 80 °C, in Millipore water, using magnetic stirring for 2 h. After the complete dissolution of the parental components, mixtures of PVA/OxC and PVA/OxP with contents of oxidized polysaccharides (expressed as weight percent) were prepared: 0.5%, 5%, 10% and 20%.

The hydrogels were obtained by freezing/thawing the aqueous solutions of pure PVA, PVA/OxC, and PVA/OxP mixtures using three consecutive cycles (16 h, freezing at −20 °C, and 8 h thawing, at room temperature), according to a method previously reported [10,11]. The resulted physical networks were dried by lyophilization. 

### 2.4. Fourier Transform Infrared Spectroscopy

A Vertex 70 spectrometer (BrukerHamburg, Germany) was used for the Fourier transform infrared (FTIR) spectra recording in the attenuated total reflection (ATR) configuration. All spectra were collected at 128 scans, at a 2 cm^−1^ resolution, in the mid IR range (4000–600 cm^−1^). The OPUS 6.5 software was used for the FTIR data processing. 

### 2.5. Nuclear Magnetic Resonance Spectroscopy

Nuclear magnetic resonance (NMR) spectra were recorded using a Bruker Avance DRX 400 MHz spectrometer. The instrument is equipped with a 5 mm QNP direct detection probe and z-gradients. Samples of pullulan, oxidized pullulan and oxidized cellulose were prepared in D_2_O using tetramethylsilane TMS (δ = 0 ppm) as an internal standard, then used to record their ^1^H-NMR and ^13^C-NMR spectra.

### 2.6. Environmental Scanning Electron Microscopy

For the morphology observation of all hydrogel samples, an environmental scanning electron microscope (ESEM, Quanta 200, FEI Company, Hillsboro, OR, USA) instrument was used. This apparatus works at 5 KV in high vacuum mode, with secondary electrons. Samples were coated with a thin layer of gold, fixed by means of colloidal silver on copper supports. 

### 2.7. Swelling Measurements

The degree of swelling (*S*, %) of samples was measured in Millipore water at 25 °C by weighting the dried samples, before (*W_d_*) and after immersing at different times (*W_t_*). After the sample immersion for a given time, this was extracted, the water excess being removed using a filter paper, and weighted in a high precision balance. All experiments were done three times, the calculations took into consideration the average values. The swelling degree (*S*, %) can be determined as:

(1) $$S\left(\%\right)=\frac{W_{t}-W_{d}}{W_{d}}\times 100$$

### 2.8. Rheological Measurements

The rheological behavior was investigated by using a MCR 302 Anton Paar rheometer (Graz, Austria) equipped with the Peltier device for the temperature control and plane geometry (the upper plate diameter of 50 mm and the gap of 500 μm). An anti-evaporation device, which realizes a saturated atmosphere near the sample, was used in order to avoid the water evaporation.

The frequency sweep tests were carried out at 25 °C in the linear domain of viscoelasticity. The self-healing was revealed at *ω* = 1 rad/s and successive strains of 1% and 400%. Creep and recovery tests were carried out for all the samples. During the creep test, different shear stress values were applied for 30 s and the evolution of deformation was followed. After the stress cessation, the recovery of deformation was registered.

### 2.9. Cytotoxicity Tests

The cytotoxicity of the selected hydrogels has been evaluated in vitro using the standard MTT test [15], on the primary fibroblast cell line at passage 5, obtained from Albino rabbit dermis, according to our previous reports [10,11]. 

### 2.10. Loading of Hydrogels With L-Arginine

The hydrogel samples were separately introduced into L-arginine solutions prepared in phosphate buffer (pH 7.4); hydrogel:L-arginine ratio, 1:1 (w/w). After 72 h stirring at 100 rpm, at 37 °C, the L-arginine loaded hydrogels were centrifuged and lyophilized. To determine the concentration of the remaining drug in supernatant, absorbance was measured. Based on the calibration curve, the percentage of L-arginine loaded in the hydrogel (*P_l_*, %) (Equation (2)) and the amount of L-arginine in each hydrogel (Equation (3)) were determined:

(2) $$P_{l}=\frac{C_{0}-C_{e}}{C_{0}}\cdot 100$$

where *C*_0_ represents the initial concentration of the drug in the feed/initial solution (µg/mL), and *C_e_* represent the drug concentration left in the solution (µg/mL).

(3) $$m_{u}=\frac{m_{0}\cdot P_{u}}{100}$$

where *m_u_* is the amount of L-arginine loaded into the hydrogel (mg), *m*_0_ is the initial amount of L-arginine used for hydrogel loading (mg), and *P_u_* is the percentage of L-arginine loaded into the hydrogel (%).

In order to establish the wavelength of L-arginine, a dilute solution of the pure drug prepared in phosphate buffer (pH 7.4) was scanned in the UV region [16]. In this way, a calibration curve could be determined. Using the pure drug, a series of seven standard solutions of known concentration (5–35 µg/mL) in phosphate buffer were prepared and the absorbance was determined at the fixed wavelength.

### 2.11. In Vitro Drug Release Studies

The experimental protocol for the in vitro drug release studies is extensively detailed in our previous study [14]. The quantitative L-arginine determination was performed using the spectrophotometric method. The amount of drug released was calculated by using the calibration curve of L-arginine, in terms of % release (Pr) as shown below:

(4) $$P_{r}=\frac{C_{e}}{C_{0}}\cdot 100,$$

where *C*_0_ represent the total concentration of the drug loaded (µg/mL), while *C_e_* represent the concentration of the released drug (µg/mL).

### 2.12. Analysis of In Vitro Drug Release Kinetics

The prediction and correlation for the in vitro L-arginine release from the modified release hydrogels was done according to the method described by Ipate et al. [17]. The experimental data from the in vitro drug release experiments were investigated using four predictable models: Zero-order, first-order kinetics, Higuchi and Korsmeyer–Peppas models [18]. The MATLAB 7.1. software (developed by The MathWorks, Inc., Budapest, Hungary) has been used for the data fitting, by linear and nonlinear regression. Data were presented as mean ± standard deviation being considered as statistically significant at p < 0.05. The correlation coefficient (R^2^) value was considered for the model determination, which best fits the release profile of each formula, whereas the “*n*” exponent gave the insight about the mechanism [19].

## 3. Results and Discussions

### 3.1. Synthesis and Characterization of Oxidized Polysaccharide Samples

Generally, cellulose applications are drastically restricted by its high recalcitrance to common solvents, including water. To overcome this issue, several chemical approaches could be designed. One convenient way to generate cellulose derivatives that are able to be processed in water, comprise of the introduction of plenty carboxyl groups which could be done by simultaneously modifying the three –OH groups in the anhydroglucose unit, as we had previously reported [11,20]. Conversely, pullulan exhibits an excellent solubility in water, being suitable for reactions in the homogeneous media [4,5,10]. The oxidation reaction performed on pullulan employing the TEMPO-mediated protocol, allow the formation of 6-carboxy pullulan, which is even more water-soluble than the original pullulan [21].

**Figure 1:**
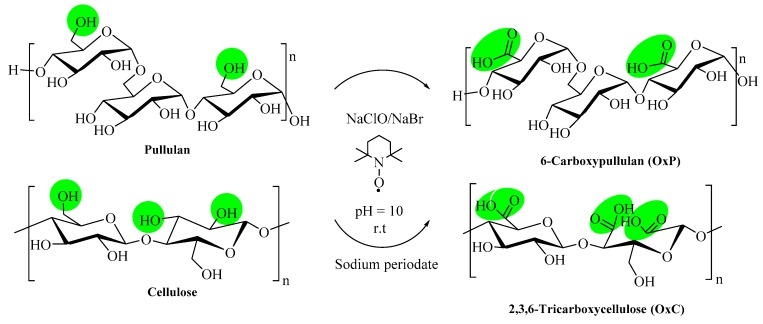
General scheme for the oxidation of pullulan and cellulose, performed in the presence of TEMPO, NaClO, NaBr and sodium periodate, respectively.

The general reaction scheme to oxidize the two primary –OH groups in the maltotriose repeating unit in pullulan, as well as the primary and secondary –OH groups in cellulose is presented in Figure 1.

The carboxylated polysaccharides were dialyzed against water, and freeze dried for further use in the hydrogel fabrication.

### 3.2. FTIR Spectra of the Composite Hydrogels

**Figure 2:**
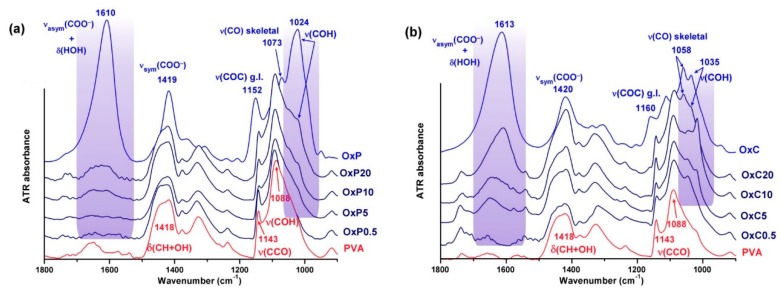
The fingerprint region of attenuated total reflection (ATR)-FTIR spectra of the poly(vinyl alcohol) (PVA) hydrogels, in comparison with the neat PVA and OxP/OxC: (**a**) OxP-PVA; (**b**) OxC-PVA.

The infrared spectra of the hydrogels with various formulations, presented in Figure 2, retained all the characteristic vibrations of the PVA matrix: The broad ν(OH) band around 3300 cm^−1^, the combined deformation band δ(CH_2_/CH + OH) peaking around 1418 and 1340 cm^−1^, and the fused ν(COH)+ ν(COC)+ δ(CCH) band in the 1200–1000 cm^−1^ region [22]. Especially for the OxC-PVA hydrogels, the symmetric stretching vibration of the carboxylate groups is clearly observed at 1610 cm^−1^ for 20% OxC loading, displaced to the red from the initial 1613 cm^−1^ value in OxC.

The compatibility among the oxidized polysaccharides and PVA can be extracted from the spectral evolution of the large ν(CO) band and here a distinction between the two types of hydrogels is observed (Figure 2a,b). The very dynamic behavior of the ν(CO) band in the OxC-PVA samples is due to the development of two new components, around 1048 and 1018 cm^−1^, even from the lowest OxC concentration. Most probably, these two bands belong to the ν(COH) vibrations of the pending OH groups (1048 cm^−1^) and to the corresponding ν(CCO) of the backbone (1018 cm^−1^), hydroxyls that are engaged in strong intermolecular interactions with the three-carboxylate groups of OxC. The redshift with 3 cm^−1^ of the ν_asym_(COO^−^) of OxC in hydrogels and the blueshift with 11 cm^−1^ of ν(OH) of PVA are additional evidences.

In OxP-PVA hydrogels, the ν(CO) band is apparently silent with the increasing concentration of OxP. But the band maximum has blueshifted from 1088 cm^−1^ in PVA to 1093 cm^−1^ in OxP-20, and there is a new component gained at 1018 cm^−1^ (ν(COH) of OxP shifted to the red with 6 cm^−1^). The ν_asym_(COO^−^) band of the OxP-PVA hydrogels is spread into a large, but weak band, centered on 1625 cm^−1^ in OxP-20, and that covers several species of carboxylates with various degrees of hydrogen bonding. As OxP is mainly amorphous, as observed from the very intense peak at 1024 cm^−1^ [23], mixing with the amorphous part of the PVA is extremely efficient, and proves an excellent compatibility among the two components.

### 3.3. Morphology of Hydrogels

**Figure 3:**
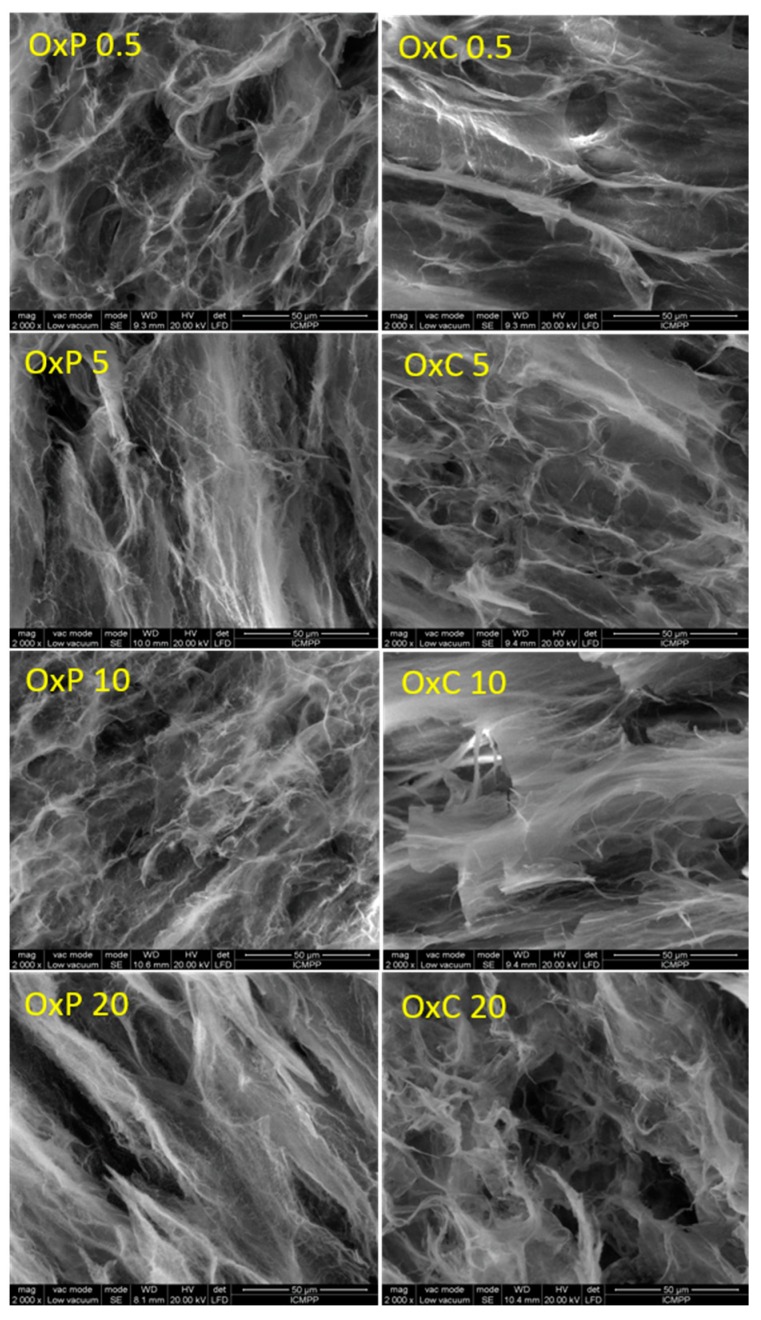
SEM microphotographs (2000× magnification) of cross-sections of PVA hydrogels containing 0.5%, 5%, 10% and 20% oxidized pullulan (OxP 0.5, OxP 5, OxP 10, OxP 20) and tricarboxy cellulose (OxC 0.5, OxC 5, OxC 10, OxC 20), respectively.

The morphology of the prepared hydrogels was analyzed by means of ESEM. As Figure 3 shows, the hydrogels prepared from PVA and oxidized polysaccharides, are porous networks with the pore size ranging from about 14 to 46 μm. All hydrogels display a homogeneous structure with no irregular areas being observed. This can be explained by the compatibility between the two components, i.e., PVA and oxidized polysaccharide. The vast porous structure with interconnected pores in all hydrogels samples, recommend them for biomedical applications, when biologically active entities can easily access the inner part of the porous network.

### 3.4. Swelling Behavior

**Figure 4:**
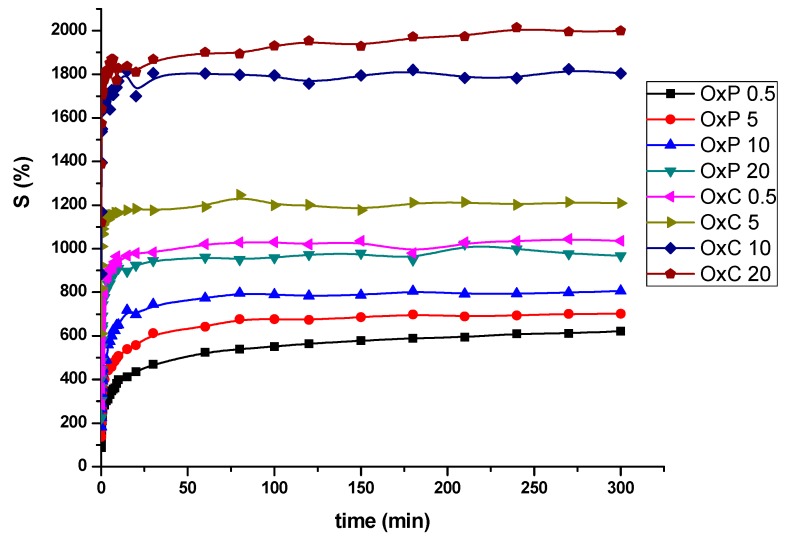
Swelling behavior of PVA hydrogels containing 0.5%, 5%, 10% and 20% oxidized pullulan (OxP 0.5, OxP 5, OxP 10, OxP 20) and tricarboxy cellulose (OxC 0.5, OxC 5, OxC 10, OxC 20), respectively.

The high amounts of hydroxyl groups originate from both PVA and oxidized polysaccharides, and also carboxyl groups in oxidized polysaccharides, have a great impact on the swelling behavior of the prepared hydrogels. The highly hydrophilic trait of the hydrogels allows the water to easily penetrate the pores of the hydrogels, Figure 4.

Even though from the SEM observations we could not find any significant differences in pore size in the case of the oxidized pullulan vs. tricarboxylated cellulose hydrogels, the swelling behavior measurements have revealed a remarkable feature, namely, the hydrogels made from PVA/OxC possess much higher swelling values than those made from PVA/OxP. Moreover, the swelling values increased, as the amount of OxC in the hydrogel increased. The higher swelling ability of the PVA/OxC hydrogels can be explained by the presence of a much higher number of hydrophilic carboxyl in these hydrogels as compared with PVA/OxP hydrogels. Another observation from SEM microphotographs can be made, namely: The pore size of the hydrogels varies with the amount of oxidized polysaccharides added during synthesis [10,11]. Larger size pores favor the swelling degree of the hydrogels, and as a consequence a higher load of drug, but at the same time, will contribute to a faster drug release [24].

### 3.5. Rheological Behavior

**Figure 5:**
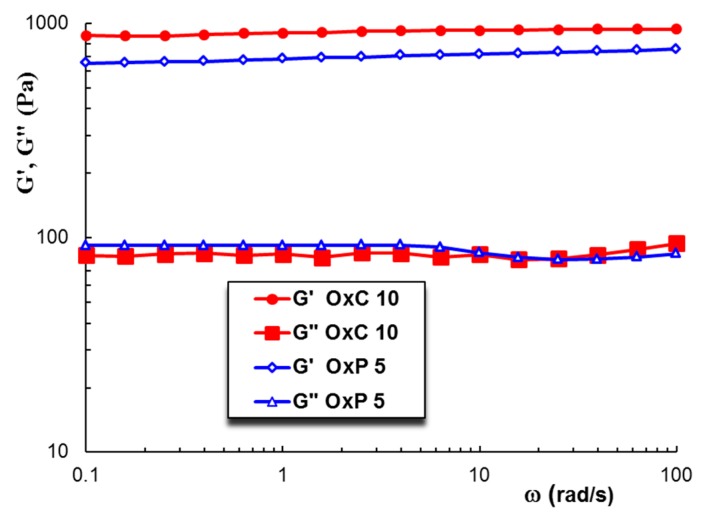
The viscoelastic moduli (G’ and G”) as a function of the oscillation frequency for hydrogels with 10% OxC (red full symbols) and 5% OxP (blue open symbols) at 25 °C (γ = 1%).

**Figure 6:**
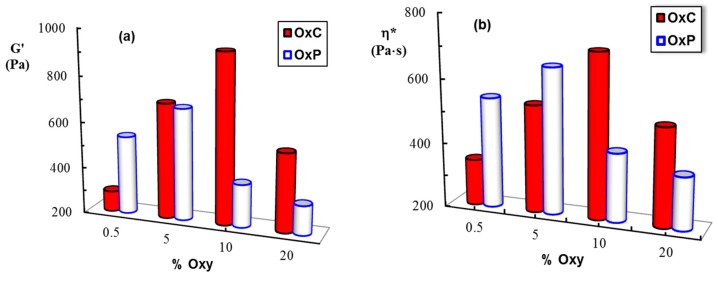
(**a**) Elastic modulus (G’) and (**b**) complex viscosity (η*) for the investigated samples with different content of oxidized polysaccharides (% Oxy) at 25 °C (ω = 1 rad/s, γ = 1%).

In frequency sweep tests, the elastic (G’) and viscous (G”) moduli were measured and these parameters give information about the reversibly stored deformation energy and the irreversibly dissipated energy during one cycle, respectively. For all investigated hydrogels, the G’ values were higher than the G” ones and they are nearly independent on ω, suggesting that the network is formed. Figure 5 shows the evolution of the viscoelastic moduli, G’ and G”, as a function of the oscillation frequency, ω, for two selected samples, OxC 10 and OxP 5, for which the highest values of viscoelastic parameters were obtained (Figure 6).

**Figure 7:**
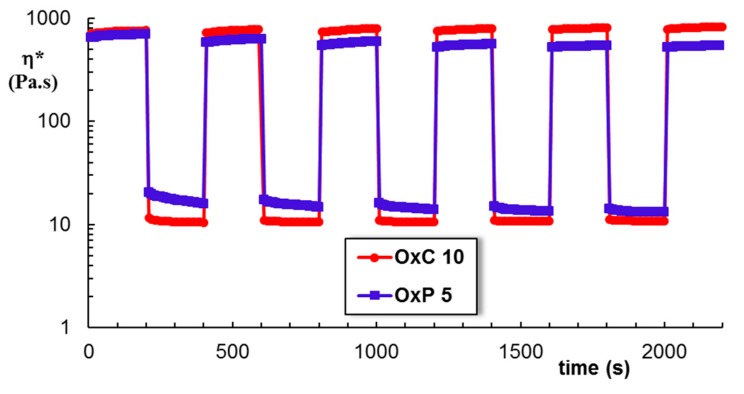
Self-healing behavior of OxC 10 and OxP 5 hydrogels when successive strains of 1% and 400% were applied (ω = 1 rad/s, 25 °C).

Very recently, we have shown that there is an optimum amount of oxidized polysaccharide for which the intermolecular interactions with PVA determine the formation of a dynamic network [10,11]. These physical hydrogels present a self-healing behavior when they are submitted to successive low (1%) and high (400%) strains, as shown in Figure 7. The structure is perturbed by applying a high shear strain and it is recovered instantaneously after healing at a low strain value, being re-stabilized through physical interactions. The rheological feature of the composite hydrogels based on PVA and oxidized polysaccharides make them of interest for wound dressing applications [25].

**Figure 8:**
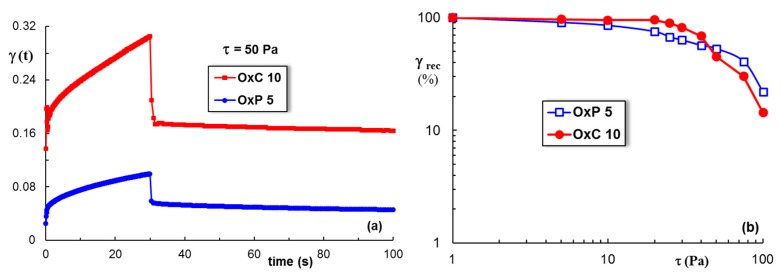
The behavior of OxC 10 and OxP 5 samples in creep and recovery tests: (**a**) A shear stress of 50 Pa was applied during the creep test; (**b**) the total elastic recovery (γ_rec_, %) as a function of the applied shear stress during creep test for OxC 10 and OxP 5 hydrogels.

During the creep test, a constant stress is applied for 30 s and the hydrogel shows a time-dependent increase in the strain (Figure 8a). The creep curves comprise the following components of the strain: The instantaneous, the retardation and the viscous parts. After the stress cessation, firstly the instantaneous part is recovered, then the retardation component, and finally it rests the viscous strain.

For shear stress up to 30 Pa, a high elasticity of hydrogels can be observed (Figure 8b), when the recovered strain (the sum of the instantaneous and the retardation components) represents more than 85% from the maximum value achieved during the creep test. Bellow 40 Pa, the OxC samples show a higher elastic recovery as compared with the OxP ones, the network is better stabilized through intermolecular interactions. Above 50 Pa, the behavior is opposite, OxP containing network is more elastic, but for all types of hydrogels the elastic recovery decreases as the shear stress exceeds this limit.

### 3.6. Cytotoxicity Assays

Several atributes are required for hydrogels in order to be applied in the tissue-engineering scaffold, such as high porosity and pore interconnectivity, which also promote the cell proliferation and differentiation. These atributes also play a crucial role for the in-flow of nutrients or vascular in-growth, and the elution of metabolic waste and biodegradation [26].

**Figure 9:**
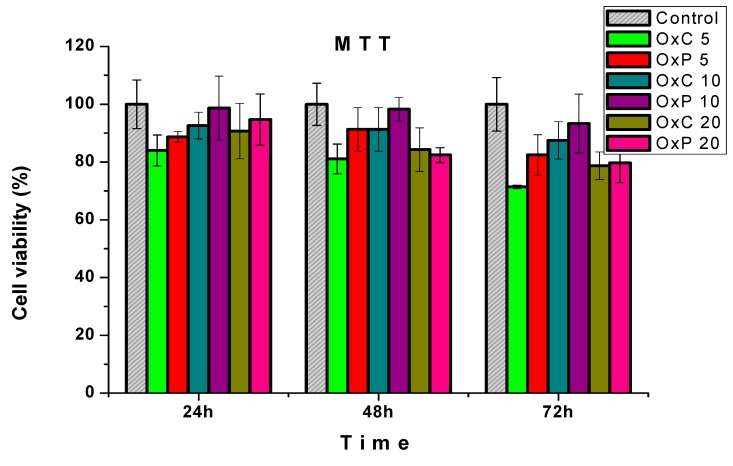
The most significant results of the cytotoxicity evaluation, performed on the PVA/carboxylated polysaccharide hydrogels by using the MTT assay.

The hydrogels prepared from PVA and carboxylated polysaccharides were in vitro cytotoxicity tested, according to the ISO 10993-5:2009 standard recommendations [15]. The results, depicted in Figure 9, reveal that all investigated samples are non-cytotoxic. As a general observation, the PVA/OxP hydrogels keep the cells metabolic activity in time. The best cytocompatibility has been found for the sample with OxP 10. In this case, after 72 h of exposure with cells, the OxP 10 sample kept cell viability over 90%. This metabolic activity of the cells culture express their ability to adapt to in the vitro conditions. The MTT results recommend these hydrogels for biomedical applications, since they do not release any cytotoxic compounds.

### 3.7. Adsorption and Release of L-Arginine

Based on the calibration curve, presented in the Supplementary Materials (Figure S1), L-arginine hydrogel loading and drug release calculations were made, Table 1.

The results of the L-arginine loading in oxidized cellulose and PVA hydrogels are consistent with the observed results in the FTIR and SEM determinations. Good dispersion of oxidized cellulose in PVA correlated with the hydrogel surface uniformity (SEM) led to a uniform loading of L-arginine in hydrogels. From the data presented, it is noted that the percentage of L-arginine increases gradually as the percentage of oxidized cellulose increases. From the SEM analysis it can be observed that there is a proportional increase between the amount of oxidized cellulose and the pore size, which allowed the diffusion of L-arginine into the porous structures of the hydrogels as the sizes increase.

The loading of hydrogels based on the oxidized pullulan and PVA is correlated with a number of physical and chemical factors. The small percentage of oxidized pullulan (0.5%) allowed a good dispersion of it in the amorphous part of the PVA matrix. The multitude of small pores (according to SEM morphology) determined an increase in the amount of L-arginine loaded on this hydrogel. As shown in the previous sections (FTIR, SEM), increasing the amount of the oxidized pullulan in the PVA matrix, will cause an increase on the optical density, resulting in a gradual decrease in drug loading, as shown in Table 1.

#### 3.7.1. In Vitro L-Arginine Release Studies

**Figure 10:**
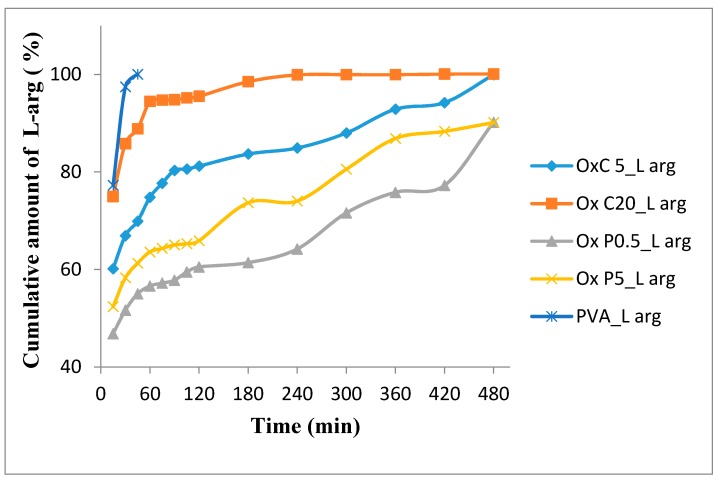
The most relevant curves showing the in vitro profile of L-arginine from the investigated hydrogels.

Figure 10 shows the release profile of L-arginine from hydrogels based on natural polymers with the modified structure and PVA at the physiological pH for normal tissue (7.4). It can be seen that the L-arginine release rate is higher for hydrogels with large pore sizes (OxC20_L-arg), as compared with those with the reduced pore size (OxP0.5_L-arg).

By associating natural oxidized polysaccharides with PVA, prolonged drug delivery systems are obtained. The hydrogel based only on PVA loaded with L-arginine, released the drug almost completely in the first 30 min (97.33% ± 0.1%), which confirms that the physical properties of PVA are not destroyed by lyophilization [27]. The morphology of the hydrogel network based on oxidized cellulose and PVA, results in a more rapid release of L-arginine due to its diffusion phenomenon from the hydrogels pores. With the increase in the amount of oxidized cellulose from the hydrogel, the amount of drug released increases. From Figure 10, it can be observed that the amount of L-arginine released by the OxC5_L-arg hydrogel, after the first 30 min, was 66.92 ± 0.51% and for the OxC20_L-arg hydrogel was 85.8 ± 0.39% and after three h the percentage increased to 83.68 ± 0.17% and 98.52 ± 0.35%, respectively. This release behavior indicates a possible physical adsorption of drug molecules to the surface of the hydrogels pores.

The release of L-arginine from hydrogels based on oxidized pullulan and PVA is directly related to the pores morphology [10]. The results of the release test show that the release rate of the drug increases with the size of the pores. Samples OxP0.5_L-arg and OxP5_L-arg, after 30 min, released 51,64 ± 0.46% respectively 58.28 ± 0.34%, and after 3 h 61,4 ± 0.61% and 73,68 ± 0.54%, respectively. The OxP0.5_L-arg sample generated a prolonged L-arginine release profile of 90.21 ± 0.15% over an eight-h period, which recommends it for use in prolonged release systems. Under these L-arginine release conditions, the OxC20_L-arg hydrogel is recommended for highly exudative wounds because this hydrogel has a high fluid uptake capacity and an increased drug release rate. Conversely, the OxP0.5_L-arg hydrogel prolonged release profile, can be related with its low swelling degree and small pore sizes [28,29]. 

#### 3.7.2. Analysis of In Vitro Drug Release Kinetics

Four drug release models, namely: Zero order, first order, Higuchi model, Korsmeyer–Peppas model, were applied to investigate the mechanism and kinetics of the in vitro release behavior of L-arginine from the hydrogels (Table 2). The correlation coefficient (R^2^) value was used to determine the model that best fits the release, and the release exponent ”*n*” gave the insight about the mechanism. The release of L-arginine from hydrogels is best described by the Korsmeyer–Peppas equation. The value of the *n* parameter, between 0.5 and 1, corresponds to a non-Fickian diffusion mechanism [30,31]. Additionally, for all samples the value for R^2^, between 0.9600 and 0.9998, suggests a release of L-arginine by diffusion. All of these data indicate that the release of L-arginine occurs by the diffusion combined with erosion/degradation of the polymer matrix of the drug [19,32].

## 4. Conclusions

In this paper, cellulose and pullulan have been selectively oxidized employing the TEMPO/NaClO/NaBr protocol, in order to introduce carboxylic groups. The resulted oxidized products were used for preparing hydrogels with PVA, employing different ratios of oxidized polysaccharides/PVA. The resulted hydrogels were investigated by using spectral and microscopic techniques and their rheological and swelling features were analyzed and further tested for the incorporation and release of L-arginine. The loading of L-arginine takes place by physical phenomena, such as diffusion, but also by chemical phenomena, when it is possible to form imine-type bonds, especially to materials with oxidized pullulan, which have a prolonged release of the drug to physiological pH. These non-invasive, L-arginine loaded materials can be used as sponges to treat heavily scarred wounds, burns or other skin conditions. In the medical field, the efficacy of these materials could be improved by introducing into the polymeric matrix some antiseptic, antimycotic and/or anti-inflammatory drugs.

## Figures and Tables

**Table 1:** L-arginine hydrogel loading.

Sample	m_hydrogel_, mg	m_L-arg_, mg	% P_u_ ± SD	m_L-arg_ Loaded, mg
OxC0.5	142	142	12.92 ± 0.51	18.34 ± 0.72
OxC5	193	193	15.32 ± 0.09	29.57 ± 0.18
OxC10	166	166	13.40 ± 0.21	22.25 ± 0.34
OxC20	201	201	16.36 ± 0.42	32.89 ± 0.84
OxP0.5	170	170	18.44 ± 0.57	31.35 ± 0.96
OxP5	178	178	15.36 ± 0.32	28.72 ± 0.59
OxP10	200	200	5.84 ± 0.40	11.69 ± 0.79
OxP20	198	198	12.48 ± 0.47	24.70 ± 0.94
PVA	205	205	11.64 ± 0.32	23.86 ± 0.65

**Table 2:** Results of curve fitting of the in vitro L-arginine release profile from hydrogels based on oxidized natural polysaccharides and PVA.

Kinetic Model	Model Coefficients	Modified Release Sample				
		OxC5_L-arg	OxC_20_L-arg	OxP0.5_L-arg	OxP5_L-arg	PVA
Zero-order	K_0_	6.1162	6.7557	5.2270	5.8099	50.12
	R^2^	0.2554	0.0842	0.4616	0.3465	0.8344
First-order	K_0_	1.5336	3.3439	0.5627	0.8223	8.8848
	R^2^	0.6919	0.9576	0.1221	0.3992	0.9789
Higuchi	K_0_	43.682	53.335	33.340	39.559	29.76
	R^2^	0.5259	0.2799	0.7224	0.6266	0.9664
Korsmeyer-Peppas	n	0.52	0.49	0.57	0.65	0.7
	K_0_	63.40	84.61	47.31	55.47	31.5
	R^2^	0.987	0.9600	0.9865	0.9832	0.9998

## Supplementary Materials

The following are available online at https://www.mdpi.com/1996-1944/12/9/1569/s1, Figure S1: Absorption spectrum.
