# Supplementary material for: Physical Hydrogels of Oxidized Polysaccharides and Poly(Vinyl Alcohol) for Wound Dressing Applications
Source: Materials (Basel). 2019 May 13;12(9):1569. doi: 10.3390/ma12091569 (PMC6539012; doi:10.3390/ma12091569)
Supplement: Supplementary file 1 [file materials-12-01569-s001.pdf]

materials

MDPI

Supplementary Materials

Physical Hydrogels of Oxidized Polysaccharides and Poly(Vinyl Alcohol) for Wound Dressings Applications

Raluca Ioana Baron, Madalina Elena Culica, Gabriela Biliuta, Maria Bercea, Simona Gherman, Daniela Zavastin, Lacramioara Ochiuz, Mihaela Avadanei and Sergiu Coseri

Adsorption and release of L-arginine

From the absorption spectrum, in the UV range, it was determined that the wavelength at which L-arginine has the maximum absorption at 202 nm.

At this wavelength the calibration curve was plotted in the concentration range between 5–35 μg/mL ($R^2 = 0.9995$).

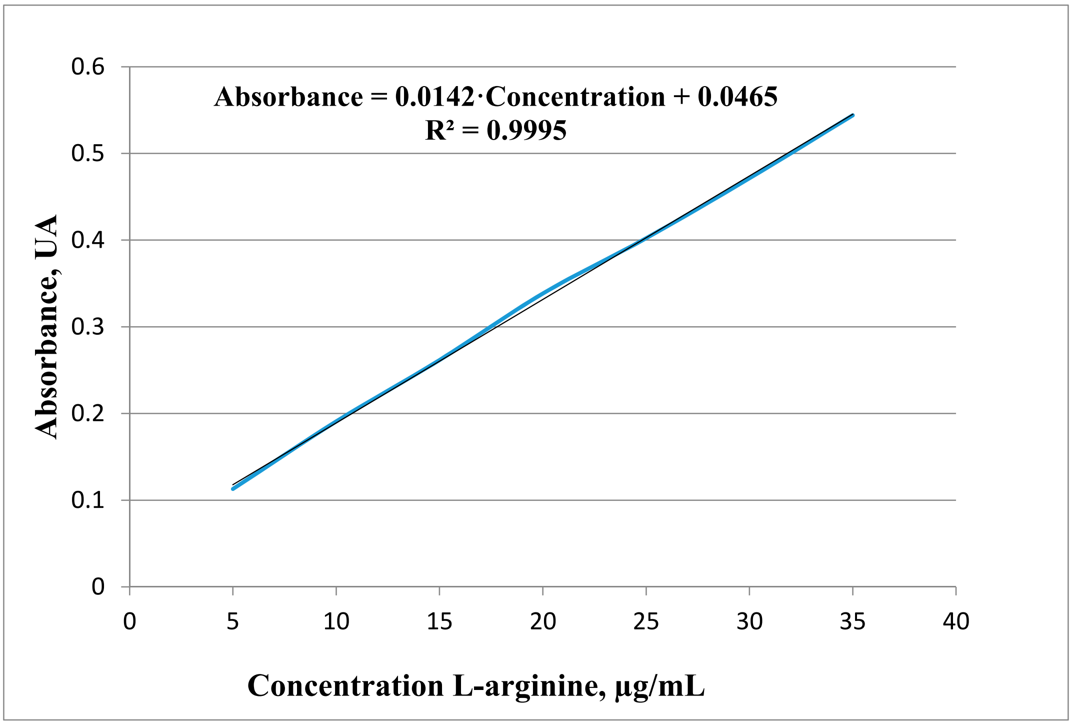

Figure S1. Absorption spectrum.

© 2019 by the authors. Submitted for possible open access publication under the terms and conditions of the Creative Commons Attribution (CC BY) license (http://creativecommons.org/licenses/by/4.0/).
